# Prediction of Bearing Capacity of the Square Concrete-Filled Steel Tube Columns: An Application of Metaheuristic-Based Neural Network Models

**DOI:** 10.3390/ma15093309

**Published:** 2022-05-05

**Authors:** Payam Sarir, Danial Jahed Armaghani, Huanjun Jiang, Mohanad Muayad Sabri Sabri, Biao He, Dmitrii Vladimirovich Ulrikh

**Affiliations:** 1 College of Civil Engineering, Tongji University, Shanghai 200092, China; payamsarir@tongji.edu.cn; 2 State Key Laboratory of Disaster Reduction in Civil Engineering, Tongji University, Shanghai 200092, China; 3 Department of Urban Planning, Engineering Networks and Systems, Institute of Architecture and Construction, South Ural State University, 76 Lenin Prospect, 454080 Chelyabinsk, Russia; danialarmaghani@susu.ru (D.J.A.); ulrikhdv@susu.ru (D.V.U.); 4 Peter the Great St. Petersburg Polytechnic University, 195251 St. Petersburg, Russia; 5 Department of Civil Engineering, Faculty of Engineering, Universiti Malaya, Kuala Lumpur 50603, Malaysia; s2005282@siswa.um.edu.my

**Keywords:** structural performance, square concrete-filled steel tube columns, metaheuristic-based ANN models, predictive models

## Abstract

During design and construction of buildings, the employed materials can substantially impact the structures’ performance. In composite columns, the properties and performance of concrete and steel have a significant influence on the behavior of structure under various loading conditions. In this study, two metaheuristic algorithms, particle swarm optimization (PSO) and competitive imperialism algorithm (ICA), were combined with the artificial neural network (ANN) model to predict the bearing capacity of the square concrete-filled steel tube (SCFST) columns. To achieve this objective and investigate the performance of optimization algorithms on the ANN, one of the most extensive datasets of pure SCFST columns (with 149 data samples) was used in the modeling process. In-depth and detailed predictive modeling of metaheuristic-based models was conducted through several parametric investigations, and the optimum factors were designed. Furthermore, the capability of these hybrid models was assessed using robust statistical matrices. The results indicated that PSO is stronger than ICA in finding optimum weights and biases of ANN in predicting the bearing capacity of the SCFST columns. Therefore, each column and its bearing capacity can be well-predicted using the developed metaheuristic-based ANN model.

## 1. Introduction

Among the concrete-filled steel tube (CFST) columns, the circular CFST (CCFST) and the square CFST (SCFST) columns have a more comprehensive range of applications and are used more often than the other types in construction as these shapes are more suitable for the concrete confinement. However, the confining action could be less efficient in SCFST column due to its angles [1,2]. Nevertheless, in current global practices, SCFST columns are also applied in the main lateral resistance systems of unbraced and braced building structures, and they also might be used for retrofitting purposes in seismic prone zones. In addition to CFST columns, this type of square infilled tube can be used as beams, caissons, and piers for deep foundations [3,4].

Some research states that concrete confinement is not efficient enough in square concrete-filled steel tube columns (SCFST) because of rigidity loss in these types of columns. In fact, in these SCFST columns, only the concrete around the center and corners of the column is restrained effectively [5]. However, various infilled concrete may behave differently to normal concrete (NC). It is of great importance that material properties can effectively influence the bonding strength between the two materials, steel and concrete [6]. In addition, the concrete’s confining degree could be enhanced by decreasing the width to thickness (w/t) ratio, while a higher value of it can cause concrete crushing as well as extra local buckling [7,8].

Several experimental and computational research were evaluated the structural performance of square concrete-filled steel tube (SCFST) columns subjected to different loading conditions or various structural parameters [8,9,10,11]. Many factors, such as the section’s size, length to width ratio, and wall thickness, were also evaluated by researchers and many design equations and formulas were generated or modified. However, the most significant factors influencing the structural behavior of the SCFST columns are the width/thickness ratio, concrete compressive strength, yield strength of the steel tube, and wall thickness [11,12,13]. Han et al. [14] investigated the behavior of the SCFST columns subjected to axial compression with a local compression area of 1.44 and 16 using experimental and numerical analysis. In total, 15 samples of SCFST columns were cast and tested in the laboratory, and they were also simulated using finite element analysis. Another study was performed by Skalomenos et al. [15] to analyze the nonlinear response of square CFSTs subjected to constant axial load. They performed a parametric study using finite element analysis to assess the expressions. The results provided the essential parameters, considering three hysteretic models, including strength and stiffness degradation. Numbers of studies were performed laboratory test for the composite columns and compared the results with the design codes in this regard such as EN 1994-1-1:2004, and GB 50936-2014. For instance, Zhang et al. [16] tested 24 composite stub columns under axial loading and compared the results with both earlier mentioned standards. In another study [17], the researchers presented a study to evaluate the grip mechanisms in infilled tube with conventional and lightweight concrete. For this purpose, they referred to different standards of AISC 360-10 and EN 1994-1-1 for the fixed value of the bond strength.

In addition to the experimental and numerical studies using finite element method (FEM), computational studies using artificial intelligence (AI) and machine learning (ML) techniques have been widely conducted in recent years. Several studies have applied artificial neural network (ANN), gene-expression programming (GEP), particle swarm optimization (PSO), imperial competitive algorithm (ICA), and many more for civil engineering applications [18,19,20,21,22,23,24,25,26,27,28]. Among them, some studies were conducted the application of ML/AI on the composite CFST columns. Jiang et al. [29] compared the results of the gene-expression programming with the finite element analysis of the circular CFST columns. Zarringol et al. [30] used ANN to predict the bearing capacity of rectangular and circular CFST columns under concentric loading conditions. Four extensive datasets were used to generate predictive models in their study. Recently, researchers developed a prediction model for the circular CFST column by using ANN, GEP, and the Adaptive Neuro-Fuzzy Inference System (ANFIS). Luat et al. [31] investigated a new methodology for predicting the bearing capacity of CCFST columns subjected to axial loading using a hybrid AI technique, which was developed based on the Bayesian additive regression tree and some optimization algorithms.

Moreover, an estimation to the compressive capacity of the CFST column was performed in another study carried out by Liao et al. [32] using fuzzy systems (FS). Their models applied firefly algorithm (FFA) and differential evolution (DE) techniques to obtain the optimum model. Another paper [33] presented an efficient ML-based framework to predict the strength of CFST columns subjected to concentric loading. The gradient tree boosting (GTB) technique was considered in their study. Their proposed framework was compared with other ML models such as tree-based models, support vector machines, and deep learning and showed more accurate results for the same purpose. 

However, among all recent research for the concrete-filled steel tube (CFST) columns using AI/ML techniques, a few research considered the square concrete-filled steel tube (SCFST) columns. Tran et al. [34] developed a study to predict the bearing capacity of the square CFST columns using the ANN technique. In their study, 300 experimental data samples were collected to be trained and tested. The trial–error method was applied to determine the optimum model in terms of the highest coefficient of determination (R^2^) and the lowest mean square error (MSE). Furthermore, many codes were adopted to assess the performance of the study. The results showed that the ANN model was more accurate than the existing formula. After validating the ANN technique, several curves were generated to accurately analyze the SCFST columns’ behavior under compressive loading. In another study [35], the short square CFST column was considered, and a comprehensive dataset was obtained by means of axial compression tests. In this research, SVM and PSO were combined to develop a new hybrid model called PSVM (SVM optimized by PSO) to predict the bearing capacity of SCFST columns. For validation purposes, the reliability of the novel model was verified against the experimental results. Le [36] proposed a model based on Gaussian Process Regression (GPR) to predict the axial load which the SCFST columns could withstand under compression, and reported a high level of accuracy for the proposed GPR model.

Furthermore, in another study [37], a GEP-based methodology is proposed to develop some equations to analyze the bearing capacity of the SCFST columns subjected to axial compression. For this purpose, six GEP-based equations were proposed. The results indicated that the proposed formulations excelled the current codes and correlations in terms of efficiency. A radial basis function neural network was applied to predict the bearing capacity of SCFST columns [38]. In this study, FFA and other optimization algorithms were also applied. A database of 300 experimental tests was collected from the literature to train the data. Several comparative criteria were also used to assess the accuracy of the proposed model. The outcomes revealed that the novel predictive model could provide a higher accuracy compared with the other similar techniques.

In structural engineering, it is essential to study the bearing capacity of the CFST columns, specifically, the SCFST column, since the grip between steel tube and concrete core in these sorts of columns is complicated, and the structural performance of these columns is often nonlinear as a result of this interaction. Furthermore, the experimental laboratory tests are generally expensive and time-consuming. In recent years, using artificial intelligence (AI) methods have become more popular as the AI approaches are usually faster and less complex in comparison with FEA. The accuracy of prediction in simulation by FE method is highly influenced by input elements which normally cannot be simulated thoroughly [39]. Therefore, using AI/ML methods for predicting the bearing capacity of the SCFST columns could be a suitable alternative. Due to the lack of research in terms of developing AI/ML techniques to predict the ultimate bearing capacity of the square CFST columns, this study aims to propose a novel technique using a combination of ANN and metaheuristic algorithms (i.e., PSO and ICA) for prediction of the ultimate axial load of these types of columns. The step-by-step modeling procedure is explained, and the obtained results are compared to select the best ANN-based metaheuristic model. 

## 2. Methods

### 2.1. Artificial Neural Network

The artificial neural network (ANN) is a method that takes advantages of a biology-based computational model that resembles the rational reactions of a human brain. ANN is a methodology for recognizing sophisticated relationships between different variables, resulting in computational models for one or a number of outputs [40,41,42]. Basically, an ANN model encompasses three fundamental components named “activating function”, “patterns of connections”, and learning rules [43]. Depending on the problem that needs to be taken into account, the components are required to be introduced to train the network considering their weights [44]. In this regard, one of the ordinary neural networks is the multilayer perceptron (MLP), which consists of a layer of input variables, one or more hidden layers of neurons’ processing, and a layer for output variables. All of these layers are connected in a sequential order, and the latter layers usually include one or more neurons together with numerical operators. Essentially, a feedforward is responsible for making signals between the output and input layers through the hidden layers. In order to specify the features of input variables, the signals, initially, have to be assessed through the hidden neurons. Later on, the specified features will be transferred to the neurons in the output layers to generate a proper model [45,46].

In recent years, various learning techniques have been suggested to improve the capability of MLP. However, backpropagation (BP) was considered a more effective method based on gradient descent [47]. This technique is benefitted from interchanging the input signs between the nodes of sequential layers. In this method, the net weight of each input “*net_j_*” is calculated as the following:

(1) $$net_{j}=\sum_{i=1}^{n}x_{i}w_{i}{}^{-\theta }$$

**Figure 1:**
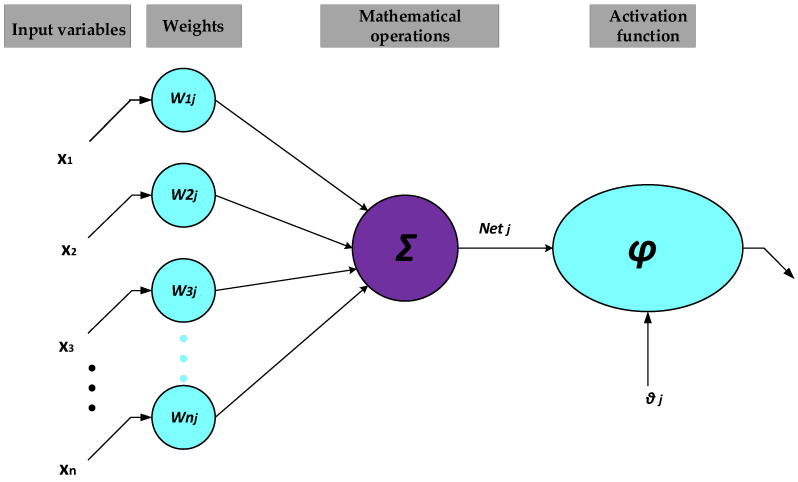
ANN structure.

In which *n* is the input’s quantity, *x_i_* is the input’s signal, and *w_i_* represents the weight of each node. Furthermore, the threshold of each node determines by the *θ* parameter. The activation functions, such as sigmoid, linear function, and step, are responsible for passing through the input variables, which is called the training step of the variables. In the next step, a comparison between the actual output and the predicted one will be made, and the discrepancy between these two can be determined [48]. Finally, the calculated errors travel back into the network to refresh the individual weights. Figure 1 indicates a numerical model for artificial neurons. During the training stage, the network behavior is assessed through proper statistical functions such as the root mean square error (RMSE) [44]. The refreshing of the weights will be continued until the system observes a decline in RMSE lower than a predefined level. The number of datasets is a significant factor in this technique, as lack of it may lead to overfitting in the training process [49].

### 2.2. Particle Swarm Optimization

**Figure 2:**
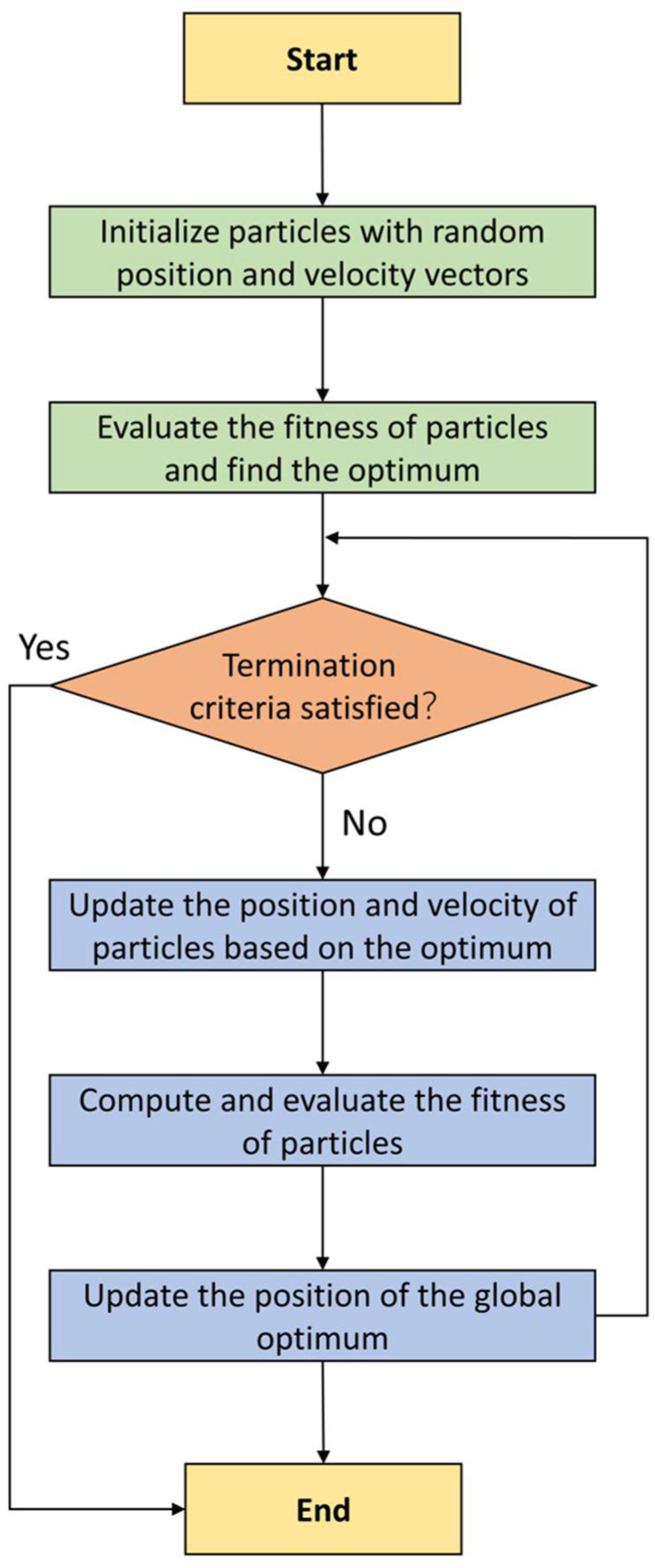
PSO algorithm to optimize problems.

In 1997 [50], Kennedy and Eberhart first proposed an intelligent computer-based technique for optimization, which later it is called particle swarm optimization (PSO). This methodology mimics the natural behavior of some creatures, such as birds, fish, bees, and ants [51]. Several algorithms later followed this technique, such as the ant colony algorithm (ACO). While some similarities exist between ACO, PSO, and genetic algorithm (GA); PSO has less complexity. In fact, PSO randomly takes advantage of the actual numbers and the relationships between the swarm particles. In the PSO technique, some entities, named particles, are distributed in an area called the objective function’s zone. The main concept in this algorithm is to locate the particles in their optimal conditions. The main elements in the particles’ movement are deterministic components and characteristics of stochasticity. In addition, they can move towards the existing global best ($p^{*})$ and also it is the best location ($x_{i}^{*}).$ Later on, the particle will look for a more suitable location compared with the previous one. In any time, “*t*”, of the specific iterations, a current best location for the *n* particles is available. Particles, finally, will look for the global best to end up with the algorithm. Figure 2 illustrates the movements of particles. As evident from the figure, $x_{i}^{*}$ is the current best for the particle *i*, and $p^{*}\approx \min\left\{f\left(x_{i}\right)\right\}$, (i=1, 2, …, n), is the global best.

Using Equation (2), given the fitness requirements for the swarms, the velocity of the swarms may be determined by a function that is proportional to the best location of the swarm and the most suitable position of each particle. Furthermore, Equation (3) leads to the successive suitable positions of the particles.

(2) $$\;\vec{v_{\mathrm{new}}}=\;\vec{v}+C_{1}\times \left(\vec{\mathrm{pbest}}-\;\vec{p}\right)+C_{2}\times \left(\vec{\mathrm{gbest}}-\;\vec{p}\right)$$

(3) $$\vec{p_{\mathrm{new}}}=\;\vec{p}+\vec{v_{\mathrm{new}}}$$

In these equations, $\vec{v_{\mathrm{new}}}$, $\;\vec{v}$, $\vec{p_{\mathrm{new}}}$, and $\;\vec{p}$ signify the new velocity, the current velocity, the new position, and the current position of particles. $C_{1}$ and $C_{2}$ shows predefined coefficients; $\vec{\mathrm{pbest}}$ is the best position of the particle itself, and $\vec{\mathrm{gbest}}$ represents the global best position among all particles. Poli et al. [52] stated that Equation (2) could be adjusted if a new parameter, inertia weight (w) is added to it. Inertia weight specifies the rate of the previous velocity of each particle to its velocity at current Equation (4). The flowchart of PSO algorithm is illustrated in Figure 2.

(4) $$\vec{v_{\mathrm{new}}}=w.\vec{v}+C_{1}\times \left(\vec{\mathrm{pbest}}-\;\vec{p}\right)+C_{2}\times \left(\vec{\mathrm{gbest}}-\;\vec{p}\right)$$

### 2.3. Imperialism Competitive Algorithm

When it comes to engineering challenges, the ICA, created by Atashpaz-Gargari and Lucas [53], is one of the most effective optimization strategies. Similar to the other techniques, ICA begins its processing by making “so-called countries” as a random initial population. After making *N* countries (*N_country_*), many of them having the lowest costs or functions, are picked up as the imperialists (*N_imp_*). As a result, colonies (*N_col_*) are specified as the remaining countries. Based on the power of empires, all colonies will be distributed to them [54,55]. Therefore, the more influential the imperialists (lowest RMSE), the more colonies can be absorbed. ICA comprises three leading operators, which are revolution, assimilation, and competition. While assimilation and revolution are in operation, a colony can reach a zone that is superior to that of its imperialist neighbor and seize control of the territory formerly controlled by the preceding imperialist.

**Figure 3:**
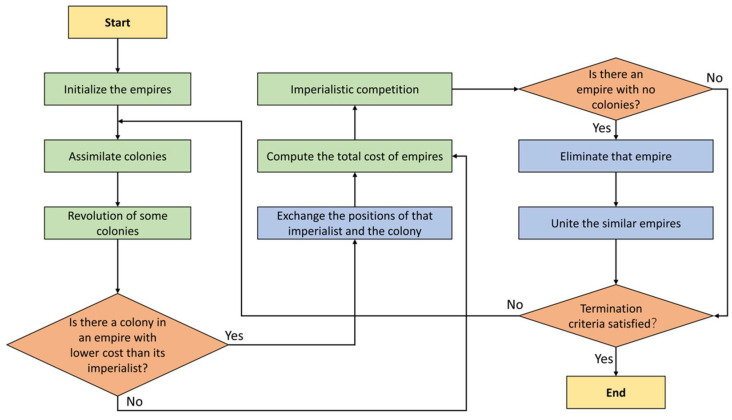
ICA algorithm to optimize problems.

However, since it is a competitive scenario, each empire has a chance to dominate at least one colony of the weakest empire, depending on the strength of the empire in question. Suppose the most powerful empire is still undefeated after a certain number of iterations or decades. In that case, the method will be repeated until a specified termination condition is satisfied, such as an acceptable RMSE, a maximum number of iterations or decades, etc. Note that the number of decades (*N_decade_*) in ICA is potentially quite comparable to the number of iterations in several other algorithms, which is worth noting [56,57]. The flowchart of ICA is shown in Figure 3.

### 2.4. Metaheuristic-Based ANN Models 

**Figure 4:**
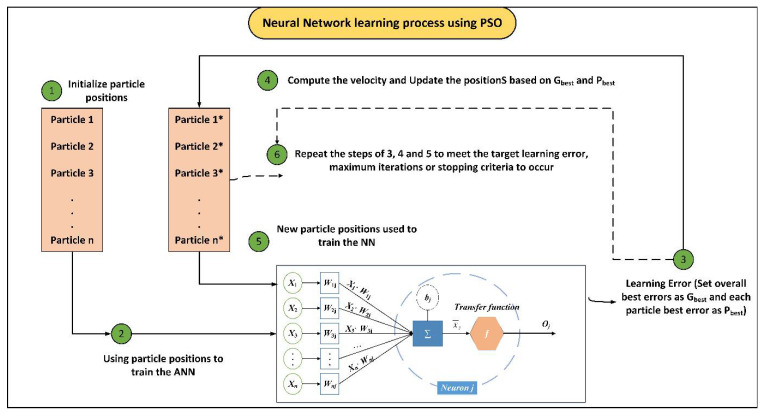
PSO-ANN algorithm.

**Figure 5:**
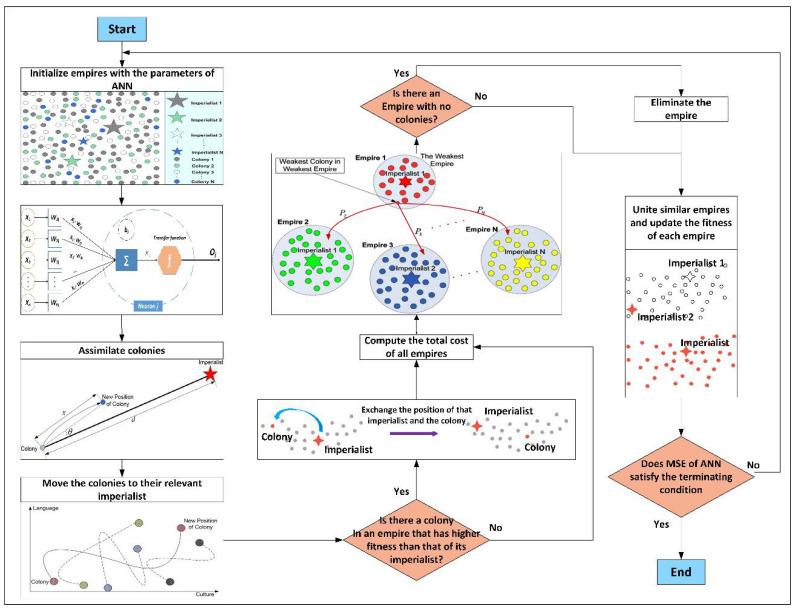
ICA-ANN algorithm.

The problem in using ANN in prediction case studies is that it will receive different results with various performance levels. It is because of the basic shortcomings of ANN, which are slow learning rate and getting trapped in local minima [58,59]. In these conditions, the possible solutions may refer to optimizing weights and biases of ANN and therefore getting more similar results by means of ANN. This optimization process can be performed by metaheuristic algorithms such as PSO and ICA. These algorithms and their effective parameters should be designed to obtain the best optimization outcome. For example, the number of countries and swarms should be designed based on a series of available range introduced in the literature. Of course, the results of hybrid models cannot differ significantly in terms of specific influential parameters. The flowcharts of hybrid models used in this study to solve the bearing capacity of the SCFST columns are presented in Figure 4 and Figure 5. As it can be seen from these figures, a number of populations (i.e., particles or countries) together with the other effective parameters of the optimization algorithms are selected and the hybrid system is trained. Then, the error indicators of the hybrid system should be measured based on the optimum weights and biases of ANN itself. Because the goal is to achieve the lowest system error possible, different values of optimization parameters can result in different system errors for the entire system. Therefore, each effective parameter should be designed using a trial-and-error system. It is worth mentioning that the base model should be designed using ANN itself. These hybrid models been applied to get more stable results in different areas of civil engineering [60,61].

## 3. Data Source and Input Parameters

### 3.1. Background

For many years, types and shapes of columns have been developed from typical reinforced concrete columns and steel columns into steel-reinforced concrete columns and various types of composite columns such as CFST columns, encased columns, and also concrete columns reinforced with concrete-filled steel tubes. Improvements and revolutions in CFST have rapidly grown during the past decades until now. Different technical journals on this topic led to the establishment of the Architectural Institute of Japan (AIJ) and a standard for circular steel and concrete, which is known as a composite structure, released in 1967. Japan and China conducted many investigations to lay the foundation for CFST later on. Then, in 1993, a study plan on composite and hybrid structures, the fifth stage of the US-Japan collaboration research program, and another study on CFST column systems were considered in the study, and the findings achieved from this investigation made the current design suggestions for the CFST column system.

**Figure 6:**
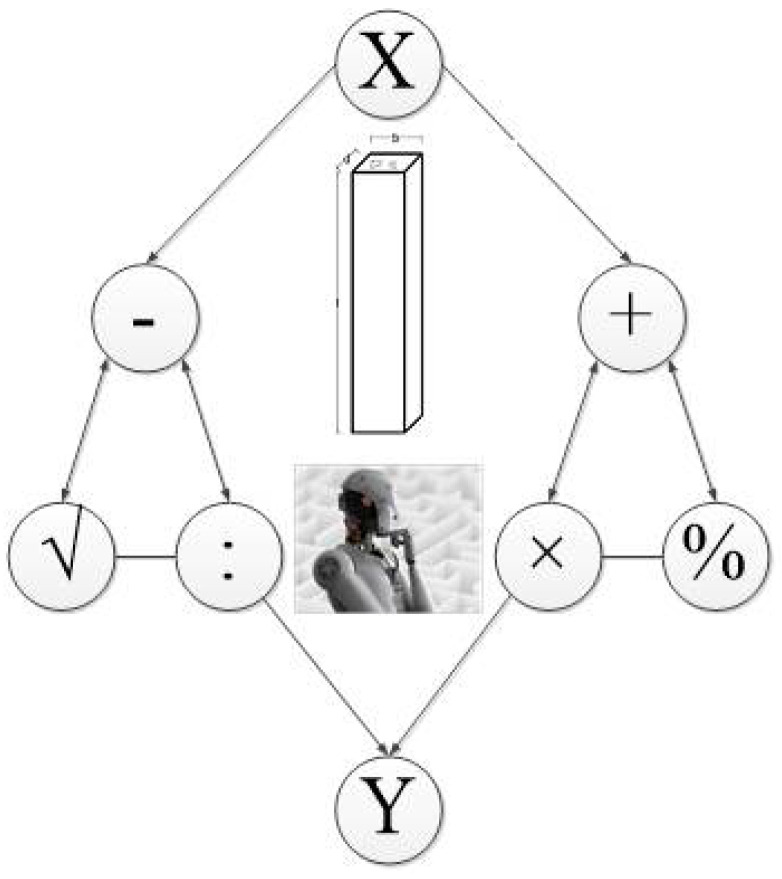
The schematic of using AI techniques for the SCFST columns.

CFST columns demonstrate more fire resistance and strength property levels than bare steel columns [62]. In addition, the filled concrete has a significant role in the structural behavior of these types of columns. High-performance concrete has superior characteristics compared to NC, for example, improved ductility, strength, and self-consolidating features. Using high-performance concrete, including lightweight concrete (LWC) and engineering cementitious concrete (ECC), could improve the ductility and strength of the concrete-filled tube composite columns. Several studies investigated self-consolidated concrete (SCC) and the NC-filled tube columns [63]. However, the current study attempts to collect comprehensive data from the literature consisting of square CFST columns with various concrete compressive strengths purely under axial compression. This limitation of datasets is due to achieving better results through prediction using ML/AI techniques. Figure 6 indicates a schematic of using ML/AI techniques for the square CFST columns.

### 3.2. Data Source 

**Figure 7:**
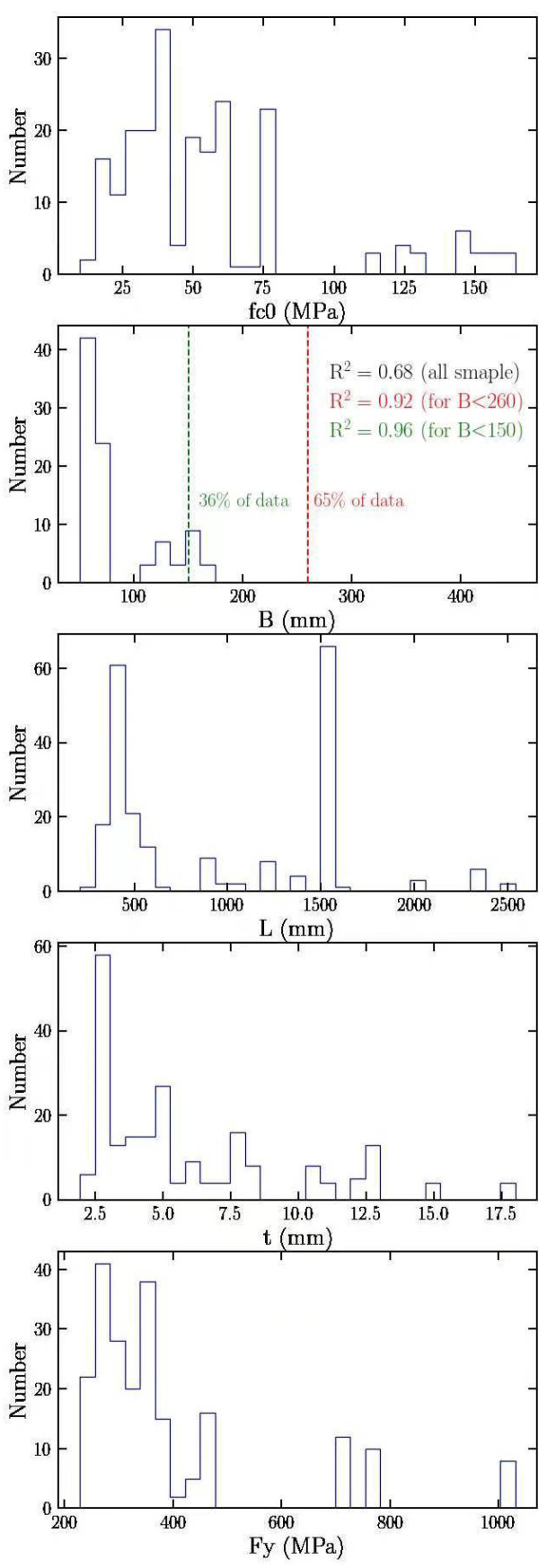
Statistical distribution of the first patch of data.

This study tries to collect extensive datasets from the literature. More than a hundred articles were studied, and among them, twenty-three articles were chosen to include their results in the dataset of this study because some of the studied articles were comprised of SCFST columns under different loading conditions or some of them with additional reinforcements inside the column, which could highly impact our result of the ultimate bearing capacity. At first, 217 data points were achieved from the articles that were taken into consideration [13,14,61,63,64,65,66,67,68,69,70,71,72,73,74,75,76]. Several essential parameters can affect analysis and results based on the previous experimental tests and experience [69,70,76,77] and some of the former data analysis for the CFST columns [26,31,35,78,79,80]. They are concrete compressive strength (*f_c_*), the width or diameter (B/D) of the columns, the length of column (L), the thickness of the columns (t), the yield strength (*f_y_*) of the steel tube, the slenderness ratio (L/D, B), and the diameter/width to thickness ratio (D, B/t). However, the most critical factors for the SCFST columns which impact the ultimate bearing capacity (*P_exp_*) were considered as five factors: concrete compressive strength (*f_c_*), the column’s width (B), the column’s length (L), the column’s thickness (t), and the yield strength (*f_y_*) of the steel tube. The minimum, maximum, average, and standard deviation values of the first patch of the collected data are shown in Table 1. The initial raw data were first analyzed considering empirical analysis and try and error. For this purpose, by taking all input parameters into consideration, the distribution of each was plotted, and later, it was considered which parameter had the most impact on the output, which was the bearing capacity, in this study. Then, considering the parameters with the dispersed data and eliminating those, it was found that which parameters had a higher impact on the R^2^. Therefore, after the preliminary analysis, it was revealed that the first patch of the collected data has a wide range of tolerance, which can cause less accuracy from the machine learning techniques. It was more critical for the wide range of the width of the columns. As shown in Figure 7, when all data were considered, the initial R^2^ was 0.68, while after the column’s width filtration to less than 260 mm and 150 mm, the R^2^ value was increased to 0.92 and 0.96, respectively, which is highly improved. It is worthy of mention that the R^2^ value is one of the significant factors in ML/AI to be considered for training and testing stages. The closer the value of R^2^ to 1, the better results can be achieved from training and testing sessions.

Therefore, it was decided to sort the data based on the preliminary results. The updated dataset led to 149 pure data points, where the range of the data was closer to each other to achieve more accurate results. Finally, the following ranges were considered in this study: 20–130.8 MPa for the concrete compressive strength (*f_c_)*, 80–450 mm for the outer width (B) of the column, 295–2340 mm for the length of the column (L), 1.94–11.25 mm for the thickness of the steel cover (t), 231–1030.6 MPa for the tensile yield stress of the steel column, and 490–3922 kN for the bearing capacities of CFST columns. The updated statistical distribution of the values is indicated in Table 2. 

**Figure 8:**
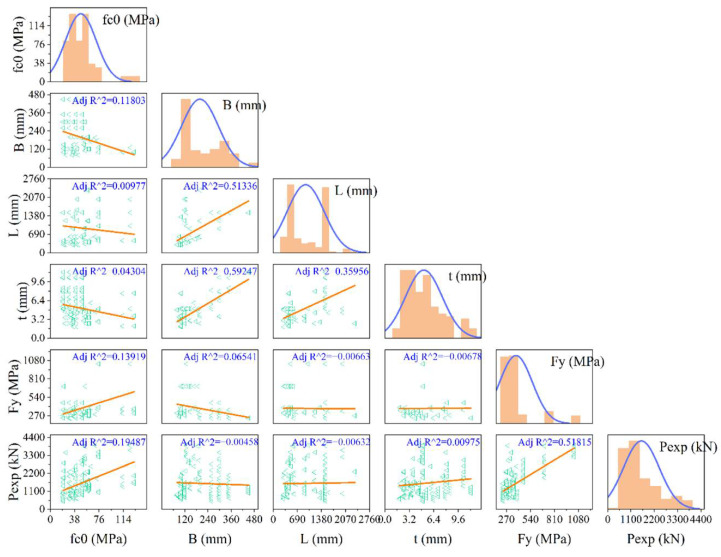
Correlation matrix for the input and output variables.

In addition, in order to show the correlation between independent variables and dependent variables, the correlation matrix was used. The correlation matrix is a matrix that can be used when several inputs can generate R^2^ (or else) with their pairs. However, it is worth mentioning that only the linear correlations between two variables can be evaluated with this approach. Therefore, it may not be capable of being used for nonlinear relationships. Figure 8 illustrates the correlations between the variables with their adjusted R^2^ values. In addition, the distributions for each parameter are presented in Figure 8. In general, the relations between variables are not that high and significant. The highest correlation between input parameters is related to the relationship of T (mm) and B (mm) with Adj R^2^ = 0.592 followed by the relationship of L (mm) and B (mm) with Adj R^2^ = 0.531. In terms of input–output relationships, *f_y_* received the highest Adj R^2^ (0.518) for predicting *P_exp_* followed by *f_c_* with Adj R^2^ = 0.195. It seems that proposing a multi-inputs model with a high level of accuracy is of importance based on these simple regression analyses. 

## 4. CFST Prediction 

The most important parameters of the ANN system should be determined before beginning the modeling steps. However, the data samples must be normalized before this can be carried out. It was recommended by Liou et al. [81] that a specific equation can be used to normalize datasets at the modeling outset in order to simplify the process:

X_normalize_ = (X − X_minimum_)/(X_maximum_ − X_minimum_)

where X_normalize,_ X_minimum_, and X_maximum_ are the normalized data sample, the minimum of each data sample, and the maximum of each data sample, respectively. 

ANN models with just one hidden layer [30,34,44] or multiple hidden layers [82,83] were presented by a number of researchers to solve various problems [45,46]. To anticipate the bearing capacity of the SCFST columns, we analyzed data from the first three layers of our data. The findings of this parametric investigation (PI) revealed that when they are compared to other implemented numbers, one hidden layer provided more accurate prediction performance than the others. ANN performance is also affected by the number of neurons in the network, which should be calculated using a different PI analysis. Preliminary research revealed that the hidden neuron values in the range of 1–11 should be considered and employed in the modeling of this section, where their root mean square error (RMSE) values were examined. As a result of these PIs, it was discovered that the hidden neuron number 9 produces bearing capacity values that are more similar to the measured values. As a result, this value was chosen as the optimal ANN model. Based on the findings from these PIs, a model with five input variables, nine hidden neurons, and one output neuron is introduced as the best ANN model, and all further hybrid modellings in this study are carried out using this model as a reference. The training and testing datasets for this investigation were selected at random from a pool of data samples representing 80% and 20% of the total number of data samples, respectively. It means that the numbers of 30 and 119 were considered for training and testing purposes of this study. 

**Figure 9:**
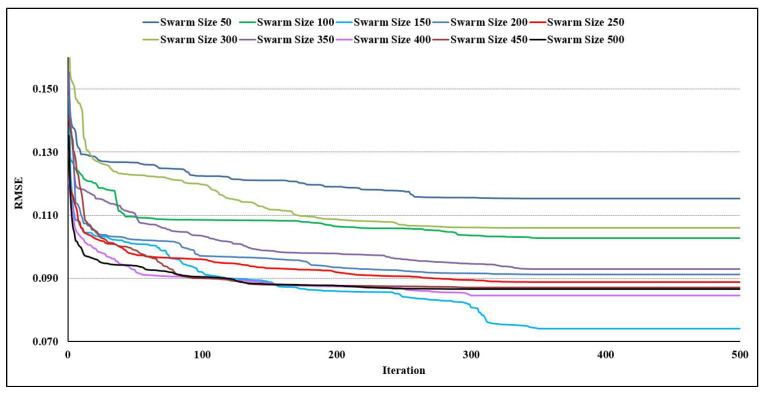
Ten PSO-ANN models and their RMSE results.

A major step in the PSO-ANN modeling process is to choose an appropriate particle size and number of iterations simultaneously during the initial stage. Through another PI, the swarm size was specified to be in the range of 50 to 500 (50, 100, 150, 200, 250, 300, 350, 400, 450, and 500). On the other hand, it was decided to set the maximum number of iterations as 500. Thus, 10 PSO-ANN prediction models were developed to forecast the bearing capacity of the SCFST columns, using the RMSE results as shown in Figure 9. As evident from the figure, the RMSE values for all of the models were significantly lowered at the beginning of the iterations. After that, the modification of the values was minimized progressively until attaining a constant value. In this manner, the situation in which the swarm size was set at 150 was the one in which the lowest error was attained. Furthermore, it can be noted that the RMSE reached a constant value after 350 rounds. As a result, to anticipate the bearing capacity of the SCFST columns for the purposes of the modeling presented in this work, the swarm size and the number of iterations used in the current article were set at 150 and 350, respectively. It is worth noting that these models were built using C_1_ = C_2_ = 2 and w = 0.25.

**Figure 10:**
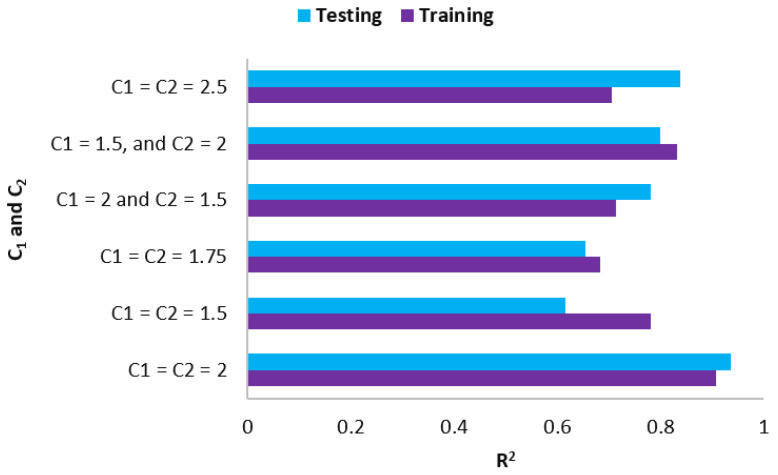
C_1_ and C_2_ results in PSO-ANN models.

On to the second step, the C_1_ and C_2_ parameters were calculated. A PI was built similarly to the previous phases, using a variety of C_1_ and C_2_ values to examine which ones were the most appropriate for our model. In order to do this, the PSO-ANN models were built using the following parameters: (C_1_ = C_2_ = 2.5), (C_1_ = C_2_ = 2), (C_1_ = C_2_ = 1.75), (C_1_ = C_2_ = 1.5), (C_1_ = 2 and C_2_ = 1.5), and (C_1_ = 1.5 and C_2_ = 2). As a factor for assessing the models’ prediction performance, R^2^ was considered. Figure 10 indicates the results in this regard. The best model was obtained with C_1_ = C_2_ = 2 as its training and testing R^2^ values are the highest among all six models shown in Figure 10. Consequently, both C_1_ and C_2_ were set to 2 and applied to the last modeling step, which was responsible for computing the “w” value. 

**Figure 11:**
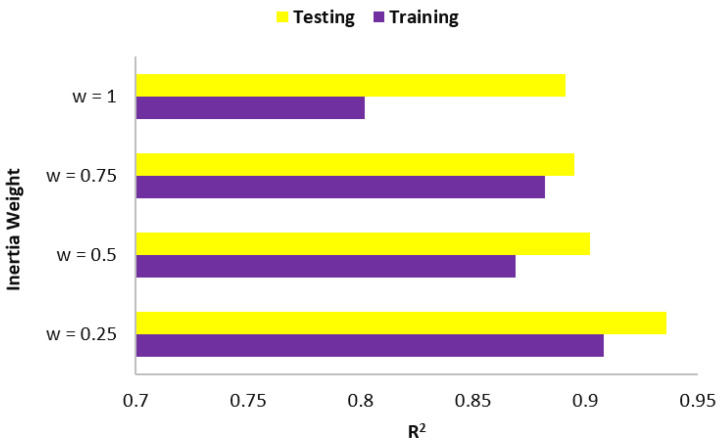
Results of w in PSO-ANN models.

The accuracy level of the PSO-ANN models is also affected by the “w” value, which can significantly influence these models [84]. As a result, in the four PSO-ANN models shown in Figure 11, the “w” value was adjusted to 0.25, 0.5, 0.75, and 1. Once again, R^2^ was selected as the performance criterion in this PI. As evident from the figure, the PSO-ANN model with w = 0.25, presented the best ability to fit and predict the bearing capacity of the SCFST columns. Therefore, as a summary for the best PSO-ANN model, the values of 150, 350, 2, 2, and 0.25 were obtained for swarm size, iteration number, C_1,_ C_2_, and w, respectively. This model will be further discussed in the next section.

**Figure 12:**
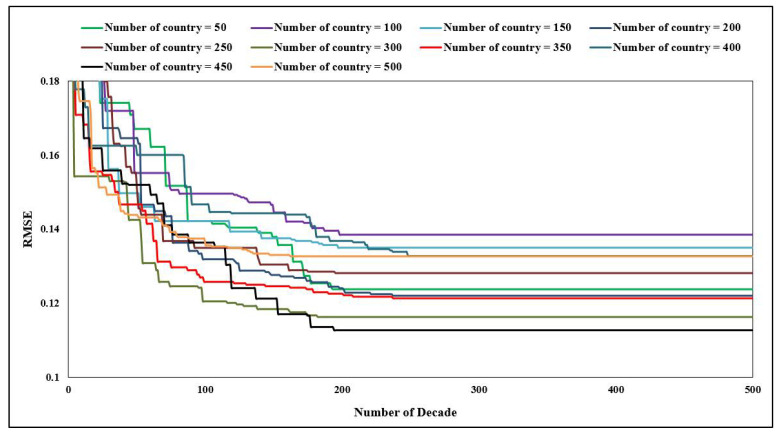
Ten ICA-ANN models and their RMSE results.

The procedures used by the ICA-ANN approach to model the bearing capacity of the SCFST columns are discussed herein. As previously stated, three factors, namely, *N_decade_*, *N_imp,_* and *N_country_* have a substantial impact on the performance capability of the ICA-ANN. As a result, designing these parameters and acquiring the optimal parameters values using various PIs is necessary to accomplish the desired results. The first PI was carried out to pick *N_decade_*, and *N_country_* at the same time. Towards to the end, the first PI was structured similarly to the preceding section and based on a variety of previously conducted research [14,50]. In order to have a fair comparison with the PSO-ANN model, the authors decided to select and use the same values of swarm for *N_country_*. As shown in Figure 12, the findings obtained from different *N_country_* are dependent on *N_decade_* that have passed and they were compared in terms of predicting the bearing capacity of the SCFST columns. As the figure simply illustrates, the majority of the countries had final RMSE values in the range of 0.11 to 0.14. When *N_country_* was limited to 450, the RMSE was reduced to its bare minimum. A further finding is that when *N_decade_* was set around 250 (almost for all *N_country_*), no further drop in RMSE was found. Therefore, the mentioned numbers were selected for these significant parameters of ICA (*N_country_* and *N_decade_*).

**Figure 13:**
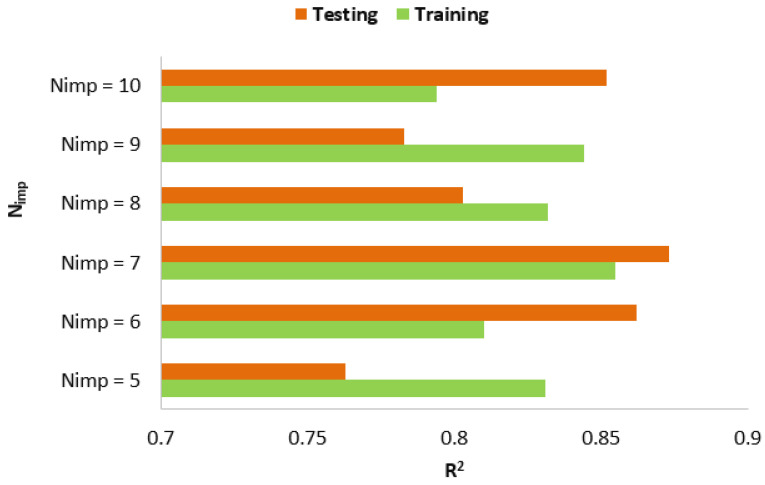
Results of *N_imp_* in ICA-ANN models.

It is now necessary to run another PI to identify the appropriate value for the amount *N_imp_*_._ According to previous studies, this was accomplished by varying *N_imp_*_._ from 5 to 10 [19,85]. The results achieved by this PI based on R^2^, are shown in Figure 13. Although the obtained results are close to each other and they are in a certain range, *N_imp_*_._ = 5 received more accurate prediction values for the bearing capacity of the SCFST columns. The R^2^ values are 0.855 and 0.873 for training and testing phases, respectively. Therefore, they were considered as the best ICA-ANN model since there are no more parameters to design. This model and its findings are discussed with further detail in the next section.

## 5. Results and Discussion

The findings of the metaheuristic-based ANN techniques in predicting the bearing capacity of the SCFST columns are addressed in this section. As previously stated, R^2^ and RMSE were used to evaluate models during their building. Another statistical index, namely variance account for (VAF), was considered and calculated for the best metaheuristic-based models. The statistical indices used in this study were widely applied in other predictive studies [21,86,87,88,89,90,91,92,93]. It is important to note that this study aims to compare the ability and power of two metaheuristic-based ANN models in predicting the bearing capacity of the SCFST columns. According to previous studies [5,7,16,94], these metaheuristic-based ANN models can achieve higher performance capacities and closer predictive values than the ANN model itself. Table 3 presents the results of statistical indices for training, testing, and all datasets of PSO-ANN and ICA-ANN models in estimating the bearing capacity of the SCFST columns. The obtained results clearly showed that PSO is the most successful model in finding the optimum values for weights and biases of ANN. This model has better performance in terms of all R^2^, VAF, and RMSE statistical indices.

**Figure 14:**
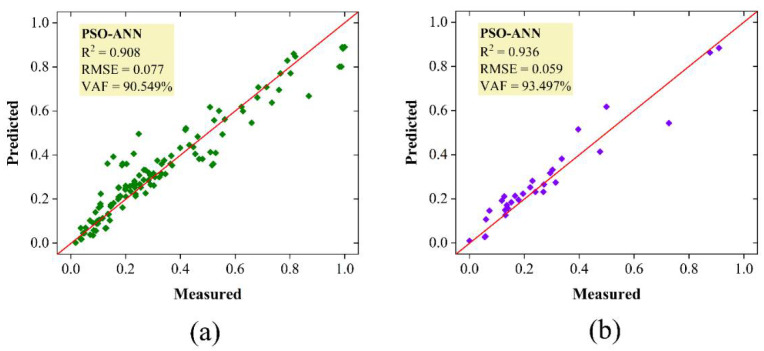
The results bearing capacity of the SCFST columns by PSO-ANN; (**a**) training, (**b**) testing.

**Figure 15:**
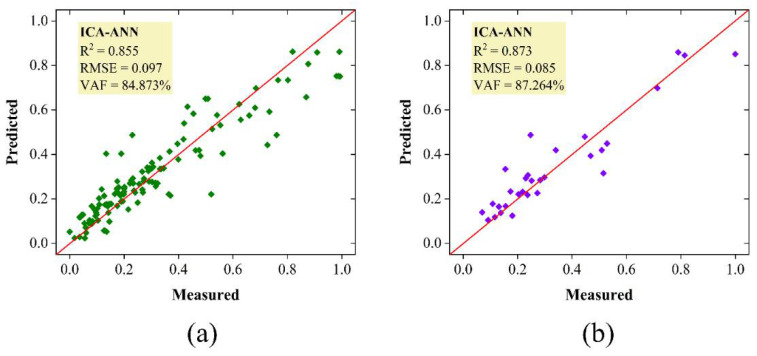
The results bearing capacity of the SCFST columns by ICA-ANN; (**a**) training, (**b**) testing.

To better understand these metaheuristic-based ANN models and their capacity for forecasting the bearing capacity of the SCFST columns, the measured and predicted values (i.e., normalized) for PSO-ANN and ICA-ANN models are displayed in Figure 14 and Figure 15, respectively. The PSO-ANN model could provide a stronger correlation between the measured and estimated bearing capacities of the SCFST columns. This model has a strong capability during the training and testing stages and, of course, for all data samples. With the anticipated and observed bearing capacities of the SCFST columns shown in Figure 14 and Figure 15, it is evident that PSO has significant potential for optimizing the weights and biases of ANN. If the weights and biases of the ANN were adequately optimized in the first place, the PSO-ANN model’s performance capabilities could be far greater than those of the ICA-ANN model. When a system error is considered, it is clear that the PSO method outperforms the ICA approach by a significant margin. Based on the above description, it is reasonable to develop the PSO-ANN model, which can get RMSE values of (0.077 and 0.059) and VAF values of (90.549% and 93.497%) for the training and testing datasets, respectively. The generated PSO-ANN technique was found to be more powerful and adaptable than the developed ICA-ANN technique in terms of solving the described issue linked to the bearing capacity of the SCFST columns.

In addition, in order to show the capability of the proposed models better, the results were compared with those obtained from the standards Euro Code 4 (EC4) and ACI Code [7,95]. Table 4 shows 30 data samples (which were randomly taken from the whole data) and their experiment bearing capacity results. In addition, predicted bearing capacity results by the PSO-ANN, ICA-ANN, EC4, and ACI are shown in Table 4. As clearly indicated, the measured results by the two metaheuristic-based ANN models are much closer than the obtained results by the standard equations. It is worth noting that for the predicted results by PSO-ANN from 30 samples, 23 data have a difference of equal or less than 10%, while for the rest of the data, the discrepancy is still less than 15%. For the ICA-ANN, the difference of the predicted bearing capacities is less than 20%. For some of the datasets, for example, datasets No. 6, 8, 9, 11, 12, 23, 26, and 31, the difference between the value obtained by the PSO-ANN method and ICA-ANN method is very small. However, the bearing capacities obtained by standards formula have a difference of 11% to 56%, which in most cases, this discrepancy is more than 20% for the models. Therefore, this indicates that the proposed metaheuristic-based models are well organized to predict the bearing capacity of the SCFST columns of the same type. 

## 6. Sensitivity Analysis

**Figure 16:**
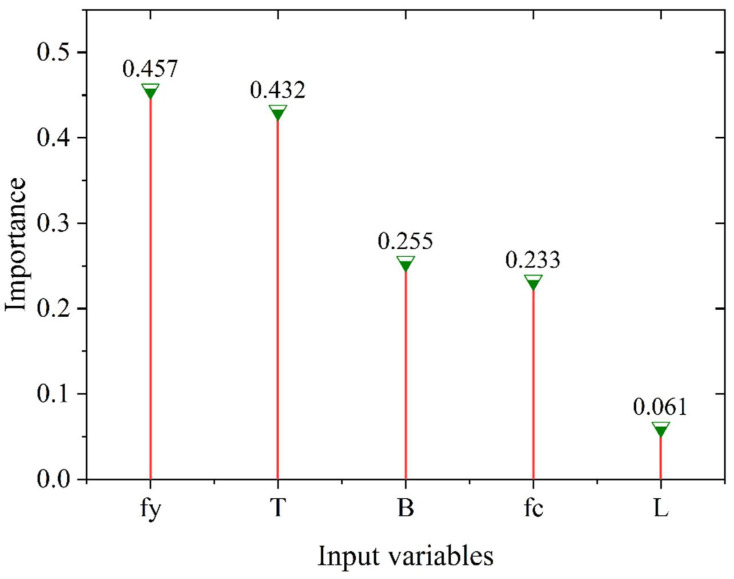
Importance of input variables.

In order to figure out the impact of the input variables (i.e., *f_c_*, B, L, T, and *f_y_*) on *P_exp_*, the mutual information (MI) method was used to analyze the importance of each variable. The MI method is a filtering method used to capture arbitrary relationships (both linear and nonlinear) between each independent variable and the target object, and thus an estimated amount of mutual information between each independent variable and the target object can be obtained [96]. Furthermore, the estimated amount lies between [0, 1]; when it is 0 then the two variables are independent and when it is 1 then the two variables are perfectly correlated. In other words, when the estimated amount is closer to 1, it means the correlation between the two variables is stronger. Based on this, the results of the relevance between these five inputs and *P_exp_* were shown in Figure 16. Intuitively, *f_y_* and T showed a significant correlation with *P_exp_*, with the respective correlation indices of 0.457 and 0.432, followed by B and *f_c_*, whose values of correlation indices with *P_exp_* are 0.255 and 0.233, respectively. As for L, it showed an insignificant correlation with *P_exp_* because of the low correlation index of 0.061.

## 7. Limitations and Future Investigations

Model generalization is one of the common limitations in concrete technology and civil engineering studies. In this study, we considered only square cases of the SCFST columns, and therefore, we used 149 data samples for modeling purposes. The proposed models are able to predict the bearing capacity of the SCFST columns if the input parameters on new data are within the range of our input parameters. Previous researchers pioneered the concept of combining theories and empirical formulas with ML/AI methods, which has been refined. 

The civil engineering communities would benefit from more investigation into this topic since a pure ML/AI model is not appealing enough to be employed. The ability to include theories and empirical formulae into the data preparation stage for a specific issue would be of the utmost importance to civil engineers at all levels. It is vital to emphasize that civil engineers do not often have enough knowledge of computer science or ML/AL models. The well-known theories and formula in this field of study may be used to create a new database, which will result in improved model performance and prediction accuracy.

## 8. Conclusions 

This research considered one of the most comprehensive databases of square SCFST columns and the bearing capacity of these columns were estimated using a series of analysis and computations. To accomplish this, the most critical parameters of the ICA-ANN and PSO-ANN models were created using a comprehensive modelling procedure. Then, their predictive ability for the bearing capacities of SCFST columns was evaluated using a variety of statistical evaluation indicators. In addition, the bearing capacities of SCFST columns were also calculated using two well-known standards and a comparison was performed. The following findings have been made as a result of this investigation:

1. Both metaheuristic-based ANN approaches performed an acceptable performance in the prediction phase, as they were able to offer values for bearing capacity close to those measured in the laboratory.
2. This study’s results indicated that the PSO-ANN model performed much better than the ICA-ANN model in both the model construction and evaluation processes. R^2^ values of 0.936 and 0.873 for PSO-ANN and ICA-ANN modelsindicate that PSO is more powerful than the ICA method at determining the optimal weights and biases of ANN.
3. The comparison between measured bearing capacities together with those predicted by metaheuristic-based ANN models as well as different standards showed that both POS-ANN and ICA-ANN models are more accurate compared to available standards. This confirmed that such intelligent techniques are needed to be used in order to obtain closer bearing capacities to the measured values.
4. Results of feature importance indicated that *f_y_* and T, with significant correlations of 0.457 and 0.432 respectively, have the highest effects on the bearing capacity of SCFST columns. Therefore, a higher level of care regarding these parameters and their designs should be considered in the laboratory while tests are planned and conducted.

## Figures and Tables

**Table 1:** Statistical distribution of data.

Parameter	Min	Max	Average	Std. Dev
*f_c_* (MPa)	10	164	55.66	35.25
B (mm)	50.8	450	208.55	106.80
L (mm)	210	2540	937.10	575.45
T (mm)	1.94	18	5.90	3.70
*f_y_* (MPa)	229	1030.60	394.60	184.25
*P_exp_* (kN)	329	8912	2139	1802.60

**Table 2:** Statistical distribution of data used in the modeling.

Parameter	Min	Max	Average	Std. Dev
*f_c_* (MPa)	20	130.8	47.70	24
B (mm)	80	450	198.50	94.90
L (mm)	295	2340	927.75	530.10
T (mm)	1.94	11.25	5.10	2.40
*f_y_* (MPa)	231	1030.60	377.20	184.60
*P_exp_* (kN)	490	3922	1588	843.70

**Table 3:** Results of training, testing, and all data samples in predicting the bearing capacity of the SCFST columns.

Metaheuristic-Based ANN Model	Training		
	VAF (%)	R^2^	RMSE
ICA-ANN	84.873	0.855	0.097
PSO-ANN	90.549	0.908	0.077
Model	Testing		
ICA-ANN	87.264	0.873	0.085
PSO-ANN	93.497	0.936	0.059
Model	Training + Testing		
ICA-ANN	85.296	0.857	0.094
PSO-ANN	91.125	0.913	0.074

**Table 4:** Comparison of the experimental results with predicted ones.

No.	P_Exp_	P_PSO-ANN_	P_ICA-ANN_	P_EC4_	P_ACI_
	(kN)	(kN)	(kN)	(kN)	(kN)
1	2275	2230	2163	2785	2520
2	1760	1625	1577	2751	2418
3	2985	2823	2738	2666	2415
4	3900	3723	3612	3441	3073
5	768	845	680	660	656
6	1426	1403	1361	1176	1059
7	1302	1445	1464	1136	1025
8	990	1007	1018	923	858
9	965	854	829	826	775
10	890	895	868	783	738
11	1530	1552	1505	1240	1127
12	1367	1355	1314	1202	1094
13	1088	971	942	940	932
14	1176	1269	1290	994	930
15	1160	1042	1011	900	851
16	1090	923	896	858	815
17	1630	1841	1890	1299	1190
18	1484	1592	1602	1262	1159
19	934	849	824	601	560
20	1934	2145	2242	1502	1477
21	2828	2995	3109	2445	2383
22	2238	2279	2300	2517	2284
23	956	1029	1070	1205	1127
24	3302.4	3450	3556	3666	3502
25	3203.8	3256	3280	3666	3502
26	3611.6	3523	3418	4383	4112
27	3474	3240	3120	4988	4695
28	840	732	702	572	553
29	860	799	775	619	593
30	1575	1592	1545	1404	1313
